# Prevalence of white spot lesions in children and adolescents across Northern, Central and Southern Italy: a multicenter cross-sectional study

**DOI:** 10.3389/froh.2026.1839880

**Published:** 2026-06-30

**Authors:** Valentina Luppieri, Flavia Iaculli, Domenica Matranga, Barbara Ravazzolo, Vera Panzarella, Giovanna Giuliana, Giuseppe Pizzo, Milena Cadenaro, Maurizio Bossù, Gianni Di Giorgio

**Affiliations:** 1 Institute for Maternal and Child Health—IRCCS “Burlo Garofolo”, Trieste, Italy; 2 Department of Oral and Maxillofacial Sciences, Sapienza University of Rome, Rome, Italy; 3 Department of Health Promotion, Mother and Child Care, Internal Medicine and Medical Specialties (Pro.M.I.S.E.), University of Palermo, Palermo, Italy; 4 Risk Management Unit, University of Palermo General Hospital (A.O.U. “Policlinico Paolo Giaccone”), Palermo, Italy; 5 Department of Precision Medicine in Medical, Surgical and Critical Care (Me.Pre.C.C.), University of Palermo, Palermo, Italy; 6 Department of Medical Sciences, University of Trieste, Trieste, Italy

**Keywords:** children and adolescents, enamel demineralization, epidemiology, oral health, prevention, risk factors, white spot lesions

## Abstract

**Background:**

Early identification of carious lesions allows the implementation of remineralization strategies, combined with improved at-home oral hygiene and dietary modifications, to reverse the demineralization process and prevent progression to cavitation. In recent years, the prevalence of white spot lesions (WSLs) in pediatric populations has increased, highlighting the importance of their monitoring.

**Objective:**

The present multicenter cross-sectional study aimed to assess the prevalence of WSLs (ICDAS-II codes 1–2) among children and adolescents in northern (Trieste), central (Rome), and southern Italy (Palermo). Additionally, the study investigated socio-demographic factors and oral health-related habits, including dietary behaviors and at-home oral hygiene practices, among participants.

**Methods:**

1,089 healthy children and adolescents were enrolled at the 3 different Department and categorized into age quartiles: ≤8; 9; 10-11; ≥12 years of age. During the first visit, patients or their caregivers were asked to complete a questionnaire including information regarding epidemiological data, family background, oral health and hygiene habits, such as toothbrushing frequency, type of toothbrush, presence of orthodontic braces, dietary habits, history of use of sweetened pacifier and frequency of dental visits in the previous year. Moreover, intraoral examinations were performed to collect tooth-level information for each patient and dental lesions classified according to International Caries Detection and Assessment System (ICDAS-II) criteria. The presence of WSLs scored as ICDAS-II 1-2 was recorded; dental caries (ICDAS-II 3-6) were also evaluated.

**Results:**

Considering the presence of WSLs, a significant difference (*p* < 0.001) was found between participants from the three Centers, with higher percentages in Palermo (66.6%) and Rome (63.1%) than in Trieste (45.9%). A statistically significant increased risk of WSLs was reported for males and 9 years old subjects and was also related to the lower parents’ working status and education. In addition, a significant enhanced risk of WSLs was associated with frequent use of sweetened pacifier, presence of orthodontic braces and high frequency of dental visits. Conversely, the risk of WSLs was lower in subjects with a frequent snack intake or in case of increased number of caries, as the lesions had already progressed to more advanced stages

**Conclusions:**

This multicenter cross-sectional study represented the first national epidemiological investigation in Italy assessing the prevalence and distribution of WSLs, along with socio-demographic factors and oral health-related habits in children and adolescents. The high prevalence observed highlights the widespread nature of the problem and the importance of early diagnosis. Early detection allows timely, non-invasive interventions to reverse enamel demineralization and prevent caries development. Promoting preventive measures and healthy lifestyle habits is therefore essential to improve quality of life and reduce the economic burden on healthcare systems.

## 1 Introduction

Dental caries is the most prevalent oral disease, representing a clear and urgent health problem worldwide (1). According to the World Health Organization's global oral health status report published in 2022, more than 2 billion people and approximately 514 million children globally suffer from untreated dental caries in permanent and primary teeth, respectively (2), with significant differences observed across countries (3). Although dental caries is a preventable disease, its negative impact on individual well-being and society, as well as its economic burden on families and healthcare systems, is substantial (1–5).

The first clinically visible sign of enamel demineralization process caused by the acidic attack of cariogenic biofilm, which, if not halted, leads to caries development, is a white, opaque, non-cavitated lesion referred to as a white-spot lesion (WSL) or, more commonly, a “white-spot” (6).

G.V. Black first described enamel WSLs in 1908 as “occasional white or ashy gray spots that were small and covered with the ordinary glazed surface of the enamel, so that an exploring tine would glide over them the same as over the perfect enamel” (7). Subsequent *in vitro* studies from the 1990s, using polarized light microscopy, confirmed the presence of an outer (pseudo) intact enamel layer covering a demineralized area with up to 25%–50% porosity, which constitutes the body of the lesion (8, 9).WSLs can develop quickly, as early as two to four weeks after dental plaque accumulation, becoming visible in their opaque, milky–white appearance when subsurface mineral loss exceeds 10% compared to healthy enamel, resulting in changes to its optical properties (10, 11). Demineralized enamel indeed has a lower refractive index (RI) and, therefore, reduced translucency compared to healthy enamel (RI = 1.66), due to the presence of air (RI = 1.00) and water (RI = 1.33) filling its increased porosities. The difference in RI between hydroxyapatite and air makes the lesion clearly distinguishable from the surrounding healthy enamel in dehydrated teeth (12–15).

Clinically, WSLs are most commonly found on the vestibular surfaces of anterior teeth, particularly along the gumline, where dental plaque tends to accumulate. In posterior teeth, WSLs typically form in the interproximal contact areas, presenting as early-stage class II lesions (14).

Since the term “white-spot lesion” refers only to the lesion color without providing information about its activity status and may be confused with other dental enamel defects, it has recently been proposed to replace it with the more accurate term “initial caries lesion” (ICL), which refers to an early non-cavitated carious lesion (16). Although initial lesions appear opaque and whitish, it should be noted that not all visible changes are necessarily initial, as they may persist for years (11, 17). WSLs can be actively undergoing demineralization, remineralization, or remain in a stable, arrested state (14, 18). According to the International Caries Detection and Assessment System (ICDAS) II, WSLs are scored in the 1–2 range (6, 19). Code 1 is characterized by the first visible change in enamel, such as opacity or discoloration, detected at the entrance of a pit or fissure after prolonged air drying. In contrast, Code 2 is defined by a distinct visual change in enamel that is visible when the surface is wet and remains visible after drying (19).

Identifying the early stages of carious lesions allows for the implementation of remineralization approaches which, combined with improved at-home oral hygiene habits and dietary modifications, aim to reverse the demineralization process and prevent its progression into cavitation (12–15, 20).

The prevalence of WSLs in pediatric patients has been increasing in recent years (14). Their occurrence is very common in patients undergoing orthodontic treatment, especially those with fixed appliances, due to plaque accumulation caused by the presence of brackets and bands which make routine oral hygiene challenging (21–23). A 2024 systematic review and meta-analysis evaluating fifty-seven studies involving 1.901 patients (mean age 16.4 years), reported a pooled prevalence of WSLs in orthodontic patients of 55.06% (24). While most epidemiological studies have traditionally focused on orthodontic patients, there is limited literature available on the prevalence of WSLs in non-orthodontic pediatric patients, particularly in primary teeth (18, 25). According to a 2022 systematic review and meta-analysis of 16 studies, the overall prevalence of WSLs in primary teeth among preschool children was estimated to be 14.0% (95% CI: 8.0–24.0) with higher values observed in low-income countries (26).

This lack of information may be attributed to the fact that the main index used in most epidemiological studies to estimate the prevalence of dental caries is the dmf-t/DMF-T (number of decayed, missing, and filled primary/permanent teeth) index, which records the individual's caries experience without distinguishing between cavitated and non-cavitated initial carious lesions, thereby underestimating thus the true presence of WSLs (27, 28). Knowing the prevalence of WSLs is particularly important for public health strategies, as it enables cost-effective and preventative approaches to managing oral health on a larger scale (26). To date, to the best of our knowledge, no epidemiological studies have been conducted to evaluate the prevalence of WSLs in italian pediatric patients on a national scale. Given the current state of the literature, the present study aimed to investigate the prevalence of WSLs (ICDAS-II codes 1–2) across Italy, specifically in northern (Trieste), central (Roma) and southern Italy (Palermo) among children and adolescents. Additionally, the study explored the socio-demographic variables and oral health-related habits (such as diet and at-home oral hygiene) among participants.

## 2 Materials and methods

### 2.1 Study design and ethics approval

The present multicentric cross-sectional study was conducted between 2022 and 2024 at the following three departments: the Unit of Paediatric Dentistry, University General Hospital of Palermo (A.O.U. Policlinico Paolo Giaccone, Palermo) (north); the Unit of Pediatric Dentistry, Policlinico Umberto I, Sapienza University of Rome, Rome (center); and the Institute for Maternal and Child Health IRCCS “Burlo Garofalo”, Trieste, Italy (south).

The study protocol which conformed to the ethical guidelines of the 1964 Declaration of Helsinki and its later amendments or comparable ethical standards, was approved by the Institutional Review Board of the University of Palermo General Hospital (A.O.U. Policlinico Paolo Giaccone; approval number 3/2022).

The paper was prepared in accordance with the Strengthening the Reporting of Observational Studies in Epidemiology (STROBE) guidelines (29).

### 2.2 Sample size calculation

The sample size for this survey was calculated to estimate the proportion of WSLs, as to guarantee a confidence level of 95% and an absolute error of 3.5%. It was assumed that the prevalence of WSLs was 14.0%, as suggested by a systematic review on primary teeth (26), assuming a clustering design with 0.05 interclass correlation. On grounds of prudence, the number of teeth per subject was assumed equal to 28, as our survey included >=12 years old children. Hypothesizing 20% of non-response, the expected sample added up to 1,065 subjects.

### 2.3 Study population

The present study enrolled children and adolescents who underwent scheduled first dental visits at the three above-mentioned departments. Parents or caregivers of all participants provided written informed consent at the first visit, allowing the use of personal data. Patients were included in the present observational study according to the following criteria: healthy subjects > 2 and < 18 years of age, absence of systemic diseases that might interfere with enamel development or morphology, absence of previous prolonged antibiotic therapies during pregnancy or in the first three years of life. Insufficient understanding of the Italian language by parents or caregivers was considered an exclusion criterion.

### 2.4 Data collection and variables

Parents or caregivers of participants were asked to complete a questionnaire including the following information: child’s age (categorized using quartiles as class breaks), gender and nationality (coded as Italian and non-Italian), family background, including parents’ marital status (coded as living as a couple and not living as a couple), mother’s and father’s educational level (categorized as compulsory education or lower education and higher education or degree), mother’s and father’s working status (coded as working or not working).

The questionnaire also included information on the child’s oral health and hygiene habits, including daily toothbrushing frequency (never, after meals, occasionally), type of toothbrush (manual, electric or no preference), orthodontic braces (no/yes), intake of sweetened foods, chewing-gum, snacks, sweetened beverages, milk and dairy products, history of use of sweetened pacifier (all coded as never/seldom or often/always), fruit and vegetables intake (once a day or twice a day/more), and dental visits in the previous year (never or once/more).

Finally, trained calibrated dental examiners (*n* = 6) performed intraoral assessments. All examinations were performed by two clinicians at each study center, all of whom were experienced in the use of ICDAS-II criteria. Prior to the study, the examiners completed a calibration exercise to standardize lesion assessment across centers. Clinical examinations were conducted under standardized conditions using artificial light, compressed air drying, dental mirrors and dental probes. The examiners collected tooth-level information for each child, including tooth position in dental arches (anterior/posterior and maxillary/mandibular), type of tooth (coded as primary incisor, primary canine, first primary molar, second primary molar, permanent incisor, permanent canine, first and second permanent premolars, first permanent molar (FPM), second permanent molar), and missing elements. For the multivariate analysis, tooth type was simplified and recoded as dentition type (primary vs. permanent) or FPM (yes vs. no). Moreover, dental lesions were identified using the modified International Caries Detection and Assessment System (ICDAS-II) criteria. The presence of WSLs scored as ICDAS-II 1-2 was recorded; the number of caries in the mouth was determined by counting the teeth with ICDAS-II scores 3–6.

### 2.5 Statistical analysis

#### 2.5.1 Patient-level analysis

The analysis followed a three-step approach: (a) descriptive and univariate analysis; (b) mediation analysis; (c) multivariate multilevel modelling. The response was the occurrence of at least one WSL in the mouth (yes/no). At univariate analysis, demographic, socio-economic, behavioural and clinical characteristics were expressed as counts and percentages for categorical variables and as mean (standard deviation) and median (range), for quantitative variables. Statistical association with the WSL outcome was assessed as odds ratios (ORs) with 95%CIs. Statistical significance was obtained from the chi-square test or the Fisher’s exact test, as appropriate, for categorical variables and from the Student’s t-test or the Wilcoxon rank sum test, as appropriate, for quantitative variables. An exploratory mediation analysis was conducted to assess whether the number of caries (M) could explain the unexpected observed association between snack intake variable (X) and the WSL outcome (Y). The analysis followed a stepwise approach: (a) estimation of the total association between X and Y, as crude OR from logistic regression; (b) estimation of the association between X and M, as IRR from a zero-inflated negative binomial regression model; and (c) estimation of the association between X and Y after adjustment for M, as adjusted OR from logistic regression model. Variables statistically significant at univariate analysis were included in the multivariate model. The observation of a reduction in the magnitude and loss of statistical significance of the association between X and Y after inclusion of M was interpreted as suggestive of a shared pathway, and the snack intake variable was carefully evaluated for inclusion in the final model. Finally, a two-level variance components logistic regression model was estimated to take into account the hierarchical data structure, with children nested within centres. Fixed effects were reported as adjusted ORs and 95%CIs, while centre-level random effects were expressed as the standard deviation of the random intercept and random intercepts, with corresponding 95%CIs.

#### 2.5.2 Tooth-level analysis

The analysis followed a two-step approach. First, a univariate analysis was performed using count-based methods (incidence rate ratios (IRRs) and the Poisson test) to explore crude associations between tooth characteristics (tooth position, dental arch and type of tooth) and the occurrence of WSL. This step allowed identification of relevant patterns and guided variable selection. Variables statistically significant at univariate analysis were included in the subsequent multivariate model. To ensure model stability and avoid sparse data issues, the type of tooth variable was represented using two binary indicators: (a) presence of first permanent molars (FPM: yes/no), and (b) dentition type (primary vs. permanent). In the multivariate analysis, the response was the presence of a WSL at the tooth level (no/yes). A three-level variance components logistic regression model was estimated to account for the hierarchical data structure (teeth nested within children, nested within centres). Fixed effects were reported as adjusted ORs (adjOR) and 95%CIs, while child- and centre-level random effects were expressed as the standard deviation of the random intercept, with corresponding 95%CIs.

## 3 Results

### 3.1 Sample description

Overall, a total of 1,089 patients aged 3–17 years were enrolled between 2022 and 2024, from three centres: Palermo (37%), Roma (43%) and Trieste (20%).

Regarding sociodemographic and behavioural characteristics, 51.8% (*n* = 564) of participants were male. Age distribution was as follows: 33.0% of the sample was ≤8 years old, 18.1% was 9 years old, 27.5% was 10–11 years old, 21.5% was ≥12 years old.

Most of the participants (84.5%, *n* = 920) were Italian, and 77.8% had a normal weight (BMI, *n* = 847). Regarding the employment status of parents, the majority were workers (84.7%, *n* = 922 for fathers; 65.5%, *n* = 713 for mothers) and had a higher education level or a university degree (65.8%, *n* = 717 for father; 59.8%, *n* = 651 for mother).

Most participants never (or seldom) used sweetened pacifier (88.2%), chewing gum (80.7%), or sugary drinks (77.5%). They ate fruit and vegetables twice a day or more (57.0%), and consumed milk and dairy products often (or always) (53.2%). Nearly half of the sample used to eat snacks often (or always) (46.6%). Most participants used a manual toothbrush (73.4%) and brushed their teeth after meals (58.2%), and 62.7% had visited a dentist once or more in the last year. Overall, patients had on average of 1.66 (SD = 2.86) decayed teeth, with a significantly higher mean in Trieste [2.68 (3.82)] than in Palermo [1.80 (2.85)] and Rome [1.06 (2.12)].

Considering the presence of WSLs, a significant difference (*p* < 0.001) was found between participants from the three Centers, with higher percentages in Palermo (66.6%) and Rome (63.1%) than in Trieste (45.9%) (Supplementary Table 1S).

### 3.2 Univariate analysis

At the Univariate analysis, there was an increased risk of WSLs for 9 years old subjects (OR: 1.74, 95% CI: 1.20–2.54, *p* = 0.003) and for males (OR: 1.5, 95% CI: 1.17–1.91, *p* = 0.001). Subjects with not working father were at higher risk of WSLs (OR: 4.93, 95% CI: 2.95–8.8, *p* < 0.001), while those with a highly educated or graduated father (OR: 0.46, 95% CI: 0.34–0.61, *P* < 0.001) or mother (OR: 0.71, 95% CI: 0.55–0.92, *P* = 0.009) were at lower risk of WSLs.

An increased risk of WSLs was associated with frequent use of sweetened pacifier (OR: 2.8, 95% CI: 1.58–5.33, *p* < 0.001) and with the presence of orthodontic braces (OR: 2.3, 95% CI: 1.5–3.64, *p* < 0.001). Conversely, a higher frequency of dental visits was associated with an increased detection of WSLs, as expected (OR: 1.6, 95% CI: 1.24–2.05, *P* < 0.001) (Table 1).

**Table 1:** Occurrence of white spots lesions related to sociodemographic and behavioral characteristics: univariate analysis.

Features	White spot lesions					
	no	yes				
	(*N* = 425)	(*N* = 664)	OR	95%CI		*P*-value
Age^a^						
less or equal to 8 years old	149 (42%)	210 (58%)				
9 years old	57 (29%)	140 (71%)	**1,74**	**1,2**	**2,54**	**0,003**
10-11 years old	110 (37%)	189 (63%)	1,22	0,89	1,67	0,219
more or equal to 12 years old	109 (47%)	125 (53%)	0,81	0,58	1,13	0,225
Sex^a^						
female	231 (44%)	294 (56%)				
male	194 (34%)	370 (66%)	**1,5**	**1,17**	**1,91**	**0,001**
BMI^b^						
normal	402 (47%)	445 (53%)				
overweight	15 (50%)	15 (50%)	0,9	0,43	1,9	0,787
obese	3 (50%)	3 (50%)	0,9	0,15	5,28	0,905
Missing	5	201				
Nationality^a^						
Italian	362 (39%)	558 (61%)				
Not Italian	63 (37%)	106 (63%)	1,1	0,78	1,54	0,616
Father occupational status^a^						
working	389 (42%)	533 (58%)				
not working	16 (13%)	109 (87%)	**4,93**	**2,95**	**8,8**	**<0.001**
Missing	20	22				
Mother occupational status^a^						
working	266 (37%)	447 (63%)				
not working	154 (42%)	212 (58%)	0,82	0,63	1,06	0,129
Missing	5	5				
Father marital status^a^						
in couple	333 (39%)	529 (61%)				
not in couple	73 (40%)	111 (60%)	0,96	0,69	1,33	0,79
Missing	19	24				
Mother marital status^a^						
in couple	333 (38%)	538 (62%)				
not in couple	88 (42%)	123 (58%)	0,86	0,64	1,17	0,345
Missing	4	3				
Father education^a^						
Compulsory education or lower	89 (27%)	244 (73%)				
Higher education or degree	318 (44%)	399 (56%)	**0,46**	**0,34**	**0,61**	**<0.001**
Missing	18	21				
Mother education^a^						
Compulsory education or lower	146 (34%)	282 (66%)				
Higher education or degree	274 (42%)	377 (58%)	**0,71**	**0,55**	**0,92**	**0,009**
Missing	5	5				
Brush teeth^b^						
never	3 (21%)	11 (79%)				
after meals	242 (38%)	392 (62%)	0,46	0,1	1,51	0,213
occasionally	180 (41%)	261 (59%)	0,41	0,09	1,36	0,153
Type of toothbrush^a^						
does not matter	29 (39%)	45 (61%)				
manual	326 (41%)	473 (59%)	0,94	0,57	1,52	0,793
electric	70 (32%)	146 (68%)	1,34	0,77	2,32	0,293
Braces^a^						
no	397 (41%)	571 (59%)				
yes	28 (23%)	93 (77%)	**2,3**	**1,5**	**3,64**	**<0.001**
Sweetened pacifier^a^						
seldom/never	402 (42%)	558 (58%)				
often/always	14 (20%)	55 (80%)	**2,8**	**1,58**	**5,33**	**<0.001**
Missing	9	51				
Chewing-gum^a^						
seldom/never	345 (39%)	534 (61%)				
often/always	78 (47%)	89 (53%)	0,74	0,53	1,03	0,074
Missing	2	41				
Fruit and vegetables intake^a^						
twice a day or more	229 (37%)	392 (63%)				
once a day	196 (42%)	272 (58%)	0,81	0,63	1,04	0,094
Snacks intake^a^						
seldom/never	197 (36%)	348 (64%)				
often/always	227 (45%)	281 (55%)	**0,7**	**0,55**	**0,9**	**0,005**
Missing	1	35				
Sweet beverages intake^a^						
seldom/never	338 (40%)	506 (60%)				
often/always	85 (41%)	120 (59%)	0,95	0,7	1,29	0,71
Missing	2	38				
Milk and diary products intake^a^						
seldom/never	175 (37%)	298 (63%)				
often/always	247 (43%)	332 (57%)	0,79	0,62	1,01	0,063
Missing	3	34				
Visits to a dentist in the year^a^						
never	187 (46%)	219 (54%)				
once or more	238 (35%)	445 (65%)	**1,6**	**1,24**	**2,05**	**<0.001**
Number of caries^c^						
Mean (SD)	2.19 (3.18)	1.33 (2.58)				**<0.001**
Median [Min, Max]	0 [0, 16.0]	0 [0, 17.0]	**0,42**	**0,3**	**0,6**	

a p-value from the Pearson's Chi-squared test;.

b p-value from the Fisher's exact test;.

c p-value from the Wilcoxon rank sum test.

Statistical significance is provided in bold.

The risk of WSLs was lower in subjects with a frequent snack intake (OR: 0.7, 95% CI: 0.55–0.9, *p* = 0.005) and decreased by increasing the number of caries (OR: 0.42, 95% CI: 0.30–0.60, *p* < 0.001). Results from the explanatory mediation analysis showed that (a) snack intake was a significant protective factor for WSLs (crude OR: 0.70, *p* = 0.005), (b) snacks intake was a statistically significant risk factor for the number of caries (IRR 1.39, *p* = 0.001) and that (c) the adjOR of WSLs related to the snacks intake, after adjustment for the number of caries was no longer statistically significant (0.79, *p* = 0.064) (data not in table). Therefore, snack intake was not included in the subsequent multivariate model due to evidence suggesting mediation through caries.

Considering tooth position and dental arch, posterior teeth (IRR 1.92, *p* < 0.001) and mandibular teeth (IRR 1.76, *p* < 0.001) were at a significantly higher risk of WSLs, with no significant differences among age classes (Table 2).

**Table 2:** Occurrence of white spot lesions (WSLs) related to tooth position, dental arch and type of tooth: univariate analysis.

Tooth-level variables	3–8 years old				9 years old				10-11 years old				more or equal to 12 years old				Overall			
	Number of teeth with WSLs *N* = 727	rate	IRR	*p*-value^1^	Number of teeth with WSLs *N* = 516	rate	IRR	*p*-value^1^	Number of teeth with WSLs *N* = 726	rate	IRR	*p*-value^1^	Number of teeth with WSLs *N* = 465	rate	IRR	*p*-value^1^	Number of teeth with WSLs *N* = 2434	rate	IRR	*p*-value^1^
Tooth position																				
Anterior	291 (40.0%)	0,08			136 (26.4%)	0,09			230 (31.7%)	0,09			164 (35.3%)	0,06			821 (33.7%)	0,08		
Posterior	436 (60.0%)	0,13	1,74	<0.001	380 (73.6%)	0,23	2,53	<0.001	496 (68.3%)	0,18	2,04	<0.001	301 (64.7%)	0,10	1,56	<0.001	1613 (66.3%)	0,15	1,92	<0.001
Dental arch																				
Maxillary	240 (33.0%)	0,07			187 (36.2%)	0,12			263 (36.2%)	0,10			181 (38.9%)	0,07			871 (35.8%)	0,08		
Mandibular	487 (67.0%)	0,14	2,02	<0.001	329 (63.8%)	0,20	1,66	<0.001	463 (63.8%)	0,17	1,72	<0.001	284 (61.1%)	0,10	1,55	<0.001	1563 (64.2%)	0,15	1,76	<0.001
Type of tooth																				
Incisor	174 (23.9%)	0,07			46 (8.9%)	0,05			90 (12.4%)	0,05			111 (23.9%)	0,07			421 (17.3%)	0,06		
Primary canine	117 (16.1%)	0,09	1,29	0,038	89 (17.2%)	0,17	3,61	<0.001	127 (17.5%)	0,17	3,21	<0.001	29 (6.2%)	0,16	2,49	<0.001	362 (14.9%)	0,13	2,15	<0.001
First primary molar	100 (13.8%)	0,08	1,15	0,276	48 (9.3%)	0,12	2,45	<0.001	34 (4.7%)	0,07	1,36	0,129	3 (0.6%)	0,03	0,44	0,224	185 (7.6%)	0,08	1,35	0,001
Second primary molar	185 (25.4%)	0,14	2,05	<0.001	178 (34.5%)	0,31	6,50	<0.001	208 (28.7%)	0,26	4,88	<0.001	40 (8.6%)	0,19	2,92	<0.001	611 (25.1%)	0,21	3,45	<0.001
Permanent canine	0 (0%)	0,00	0,00	-	1 (0.2%)	0,05	1,10	<0.001	12 (1.7%)	0,07	1,36	0,297	24 (5.2%)	0,04	0,54	0,005	37 (1.5%)	0,04	0,69	0,032
First permanent premolar	1 (0.1%)	0,07	0,94	1,000	1 (0.2%)	0,02	0,33	<0.001	29 (4.0%)	0,08	1,53	0,052	50 (10.8%)	0,07	1,05	0,797	81 (3.3%)	0,07	1,13	0,343
Second permanent premolar	0 (0%)	0,00	0,00	-	2 (0.4%)	0,12	2,46	0,082	7 (1.0%)	0,05	0,93	1,000	55 (11.8%)	0,08	1,29	0,140	64 (2.6%)	0,08	1,27	0,078
First permanent molar (FPM)	148 (20.4%)	0,21	3,04	<0.001	145 (28.1%)	0,25	5,24	<0.001	212 (29.2%)	0,23	4,30	<0.001	121 (26.0%)	0,14	2,14	<0.001	626 (25.7%)	0,20	3,31	<0.001
Second permanent molar	1 (0.1%)	0,17	2,36	0,346	5 (1.0%)	0,71	14,94	-	5 (0.7%)	0,11	2,14	0,094	32 (6.9%)	0,07	1,11	0,606	43 (1.8%)	0,09	1,40	0,042
Missing	1 (0.1%)				1 (0.2%)				2 (0.3%)				0 (0%)				4 (0.2%)			

1 Poisson test for the ratio between two rate parameters: posterior (F), inferior (S), incisive (T) as reference categories.

The occurrence of WSLs was significantly higher in canines, first and second primary molars and FPMs. Nine-year-old children exhibited the highest IRRs for all types of teeth, except permanent canine and first premolars (Table 2).

### 3.3 Multivariate analysis

Based on the two-levels variance components model, an increased risk of WSLs was confirmed for males (adjOR: 1.44, 95% CI: 1.10–1.89, *p* = 0.009) and for subjects with a non-working father (adjOR: 2.61, 95% CI:1.42–4.82, *p* = 0.002), whereas a decreased risk was observed for patients with a highly educated or graduated father (adjOR: 0.52, 95% CI: 0.35–0.79, *p* = 0.002). Regarding eating habits, an increased risk of WSLs was confirmed in association with frequent use of sweetened pacifiers (adjOR: 2.12, 95% CI: 1.08–4.16, *p* = 0.028). A higher risk of WSLs was also found in subjects who had at least one dental visit in the last year (adjOR: 1.43, 95% CI: 1.07–1.91, *P* = 0.016) and in those with a lower number of caries (adjOR: 0.82, 95% CI: 0.87–0.96, *P* < 0.001). Unobserved heterogeneity across centres was statistically significant [SD of the random intercept = 0.29, 95% CI = (0.11–0.93)] (Table 3).

**Table 3:** Children with white spot lesions related to sociodemographic and behavioral characteristics: Two-levels variance components model.

Sociodemographic and behavioral characteristics	OR	95%CI	*P*-value
*Schoolchildren level*			
Age			
9 years old	1,24	0.81, 1.91	0,318
10–11 years old	1,02	0.71, 1.46	0,929
more or equal to 12 years old	0,80	0.55, 1.17	0,245
Sex			
male	**1,44**	**1.10, 1.89**	**0,009**
Father occupational status			
not working	**2,61**	**1.42, 4.82**	**0,002**
Father education			
Higher education or degree	**0,52**	**0.35, 0.79**	**0,002**
Mother education			
Higher education or degree	1,18	0.80, 1.73	0,400
Braces			
yes	1,62	0.97, 2.69	0,064
Sweetened pacifier			
often/always	**2,12**	**1.08, 4.16**	**0,028**
Visits to a dentist in the year			
once or more	**1,43**	**1.07, 1.91**	**0,016**
Number of caries	**0,92**	**0.87, 0.96**	**<0.001**
*Random effects*			
Centre: standard deviation of the random intercept	0,29	0.11–0.93	

Reference categories for ORs: female (sex), less or equal to 8 years old (age), working (Father and Mother occupation), Compulsory education or lower (Father and Mother education), no (Braces), seldom/never (Sweetened pacifier, Snacks), never (Visits to a dentist).

Statistical significance is provided in bold.

**Figure 1:**
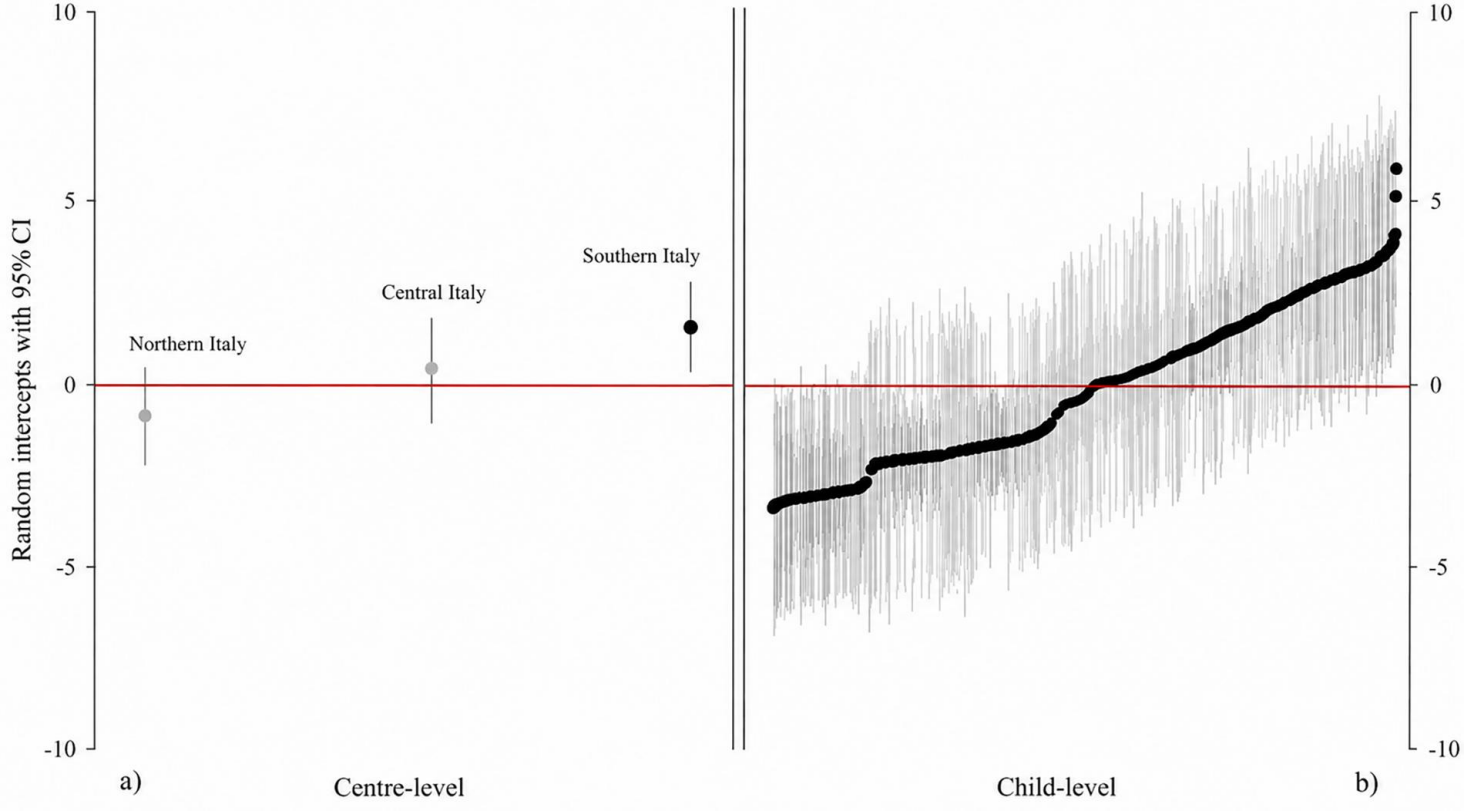
Three-level variance component logistic regression model for the occurrence of white spot lesions. Panel **(a)** shows the three centre-level random intercepts for Northern, Central, and Southern Italy, indicating a North-South gradient. Panel **(b)** displays the 1,089 child-level random intercepts, revealing substantial unobserved heterogeneity among children. Confidence intervals that do not cross the zero reference line indicate statistically significant centre-level or child-level effects.

According to tooth position and dental arch, posterior teeth (adjOR: 1.51, 95% CI:1.34–1.71, *p* < 0.001), teeth in the mandibular dental arch (adjOR: 2.09, 95% CI:1.88–2.31, *p* < 0.001), FPMs (adjOR:2.43, 95% CI: 2.05–2.89, *p* < 0.001), and primary teeth (adjOR: 1.77, 95% CI: 1.52–2.07, *p* < 0.001) were at a significantly greater risk of WSLs. Unobserved heterogeneity was also significant at the centre-level [SD of the random intercept = 1.13, 95% CI = (0.58–3.38)] as well as at child-level [SD of the random intercept =3.04, 95% CI = (2.79–3.98)] (Table 4, Figure 1).

**Table 4:** White spot lesions related to teeth characteristics: three-levels variance components model.

Teeth characteristics	OR	95%CI	*P*-value
*Tooth level*			
Position			
Posterior	1,51	1.34, 1.71	**<0**.**001**
Dental arch			
Mandibular	2,09	1.88, 2.31	**<0**.**001**
Presence of First Permanent Molar (FPM)			
Yes	2,43	2.05, 2.89	**<0**.**001**
Dentition type			
Primary	1,77	1.52, 2.07	**<0**.**001**
*Random effects*			
Child: standard deviation of the random intercept	3,04	2.79; 3.98	
Centre: standard deviation of the random intercept	1,13	0.58; 3.38	

Reference categories for ORs: Anterior (Position), Maxillary (Dental arch), No (Presence of FPM), Permanent (Dentition type).

Statistical significance is provided in bold.

## 4 Discussion

Enamel demineralization represents a relevant public health concern, contributing to the onset of WSLs which, in the absence of appropriate treatment, may rapidly progress to cavitation (20). Due to the presence of multiple risk factors, a high prevalence of enamel demineralization has been reported among the pediatric population (30, 31). In this context, the establishment of national-level epidemiological data may play a central role in developing evidence-based treatment guidelines, with potential benefits for caries prevention, oral health maintenance and the economic burden on both public and private healthcare systems. Therefore, the present multicenter cross-sectional study aimed to evaluate the prevalence of WSLs (ICDAS-II codes 1–2) in children and adolescents attending three public dental care departments in northern (Trieste), central (Roma), and southern Italy (Palermo). A significantly higher prevalence of carious lesions (ICDAS-II codes 3–6) was observed in Trieste (north) compared with the other two centers. The overall prevalence of WSLs was 60,9%; specifically, WSLs were significantly more prevalent in Palermo (66.6%) (south) and Rome (63.1%) (center) than in Trieste (45.9%) (north). This trend supports the concept that a higher prevalence of dental caries is related with a lower prevalence of WSLs, probably because the lesions have already progressed to more advanced stages. Children and adolescents enrolled in the present study were characterized by a high frequency of inappropriate dietary habits, such as the use of sweetened pacifiers (88.2%), chewing gum (80.7%) and sugary drinks (77.5%), with remarkable differences across the centers.

Regarding the lack of preventive strategies, most of the enrolled participants (73.4%) used manual toothbrushes and just over half reported brushing after meals. Although Chicalé-Ferreira et al. reported that rotational electrical toothbrushing increased wear on enamel of primary teeth affected by WSLs (32), manual toothbrushing appeared to be less effective than electric brushings in reducing plaque accumulation in primary and mixed dentition (33).

In the present study, age (9-year-old children), the presence of braces and snack intake were found statistically significant at the univariate analysis, even if these associations were not confirmed in the multivariate models. In our sample, the presence of orthodontic braces was associated with an increased risk of WSL, consistent with Hussain et al. that reported a pooled prevalence of WSLs of 55.06% in 1.901 orthodontic patients (mean age 16.4 years) (24). However, this association was not confirmed at multivariate analysis suggesting that it may be mediated by other factors, such as oral hygiene behaviours and individual baseline risk (34). Therefore, implementing appropriate preventive strategies before the initiation of orthodontic treatment could play a key role in reducing the incidence of WSLs during therapy (35). Finally, the unexpected inverse association between snacks intake and the occurrence of WSL could be explained by the presence of caries. Rather than indicating a true protective effect, this finding may reflect different stages of the caries process, whereby children with more advanced lesions are less likely to present early manifestations of the disease, such as WSLs. However, given the cross-sectional design of the study, it was not possible to establish causal pathways or mediation effects among WSLs, caries experience and snack intake. Therefore, these findings should be considered exploratory and interpreted as an attempt to better understand the interplay among factors involved in the same disease process.

Greater emphasis should be placed on the adjusted results from the multivariate analysis, which provides a more reliable estimate of the independent associations with the outcome. Specifically, participants attending at least one dental visit per year showed a higher probability of WSLs, whereas the risk decreased with an increasing number of carious lesions. These findings highlighted the need to schedule more frequent follow-up visits to detect lesions at an early stage, when remineralization is still possible, and to implement individualized preventive strategies. Furthermore, parental supervision plays a crucial role during childhood and adolescence, significantly influencing oral health habits and attitudes. Parents represent not only an example of health behaviors and sources of positive reinforcement for proper hygiene practices, but also influence dietary habits and exposure to health-promoting environment (36). In this regard, social inequalities are strongly related with oral health (1), consistent with our findings showing a significantly lower prevalence of WSLs among children whose parents had a higher level of education. Conversely, increased risk of WSLs was confirmed for males and for subjects with a non-working father. Moreover, multivariate analyses reported a significantly increased risk of WSLs in association with frequent use of sweetened pacifiers, highlighting the tight link between dietary habits and development of initial caries. Finally, the occurrence of WSLs was also related to teeth characteristics. In the studied population deciduous teeth were more frequently affected. These findings may be explained by several risk factors, as primary enamel is less mineralized and therefore more susceptible to demineralization (37). The greater frequency of WSLs observed in the mandibular arch, posterior teeth and in presence of the FPM, may be related to the increased susceptibility to plaque accumulation and cleaning difficulties as well as to the eruption stage of the FPM, that might be positioned below the occlusal plane (14).

While the present study provides helpful insights into the relationship between WSLs and various risk factors, the interpretation of the results requires some consideration. A detailed comparison with the existing literature on the prevalence of WSLs across different age groups was not feasible, due to the marked heterogeneity in sample characteristics and data collection methodologies among published epidemiological studies. Moreover, as the study was performed in dental practice settings in specific locations, the generalizability of the findings to other populations may be limited. However, it should be noted that only patients attending the three participating departments for scheduled first dental visits were enrolled, in order to minimize potential bias in the study outcomes. The cross-sectional design did not allow causal relationships between variables, limiting the interpretation of temporal associations. In addition, potential differences among participating centers might have influenced data collection and clinical assessment despite the use of standardized procedures and calibrated examiners. Finally, the limited information regarding measurement reliability, although calibration procedures were performed before the beginning of the study, might be considered as an additional limit. However, to the best of our knowledge, this is the first nationwide cross-sectional study aimed at investigating the prevalence of WSLs, as well as the associated socio-demographic variables and oral health-related habits in Italy. The multicenter design increased the heterogeneity and representativeness of the study population and the relatively large sample size strengthened the robustness and reliability of the analyses. Furthermore, the integration of clinical examination findings with questionnaire-based information enabled a more comprehensive assessment of the variables under investigation, contributing to a more comprehensive understanding of the multifactorial nature of WSLs. Future research should focus on extending the study to multiple centers, allowing for a broader assessment of the prevalence of WSLs across Italy, potentially incorporating community-based assessments, such as those conducted in schools.

## 5 Conclusion

This multicenter cross-sectional study is the first epidemiological study conducted on a national level in Italy and provides a picture of the prevalence and distribution of WSLs, as well as of the socio-demographic variables and oral health-related habits in children and adolescents. The high prevalence of WSLs observed in the study population highlights the widespread and significant nature of the problem, emphasizing the importance of early diagnosis. Early detection of WSLs is therefore essential, as it enables timely, non-invasive interventions aimed at reversing the enamel demineralization process that can lead to caries development, and at remineralizing the affected enamel while the lesions are still in a reversible stage. Implementing preventive measures and promoting healthy lifestyle habits are essential to improving the overall quality of life, particularly among children and adolescents, while helping to reduce the economic burden on both public and private healthcare systems.

## Data Availability

The raw data supporting the conclusions of this article will be made available by the authors, without undue reservation.
