# Correction: Prevalence of white spot lesions in children and adolescents across Northern, Central and Southern Italy: a multicenter cross-sectional study

**DOI:** 10.3389/froh.2026.1938378

**Published:** 2026-07-22

**Authors:** Valentina Luppieri, Flavia Iaculli, Domenica Matranga, Barbara Ravazzolo, Vera Panzarella, Giovanna Giuliana, Giuseppe Pizzo, Milena Cadenaro, Maurizio Bossù, Gianni Di Giorgio

**Affiliations:** 1 Institute for Maternal and Child Health—IRCCS “Burlo Garofolo”, Trieste, Italy; 2 Department of Oral and Maxillofacial Sciences, Sapienza University of Rome, Rome, Italy; 3 Department of Health Promotion, Mother and Child Care, Internal Medicine and Medical Specialties (Pro.M.I.S.E.), University of Palermo, Palermo, Italy; 4 Risk Management Unit, University of Palermo General Hospital (A.O.U. “Policlinico Paolo Giaccone”), Palermo, Italy; 5 Department of Precision Medicine in Medical, Surgical and Critical Care (Me.Pre.C.C.), University of Palermo, Palermo, Italy; 6 Department of Medical Sciences, University of Trieste, Trieste, Italy

**Keywords:** children and adolescents, enamel demineralization, epidemiology, oral health, prevention, risk factors, white spot lesions

The funder Italian Ministry of Health through the contribution given to the Institute for Maternal and Child Health IRCCS Burlo Garofolo (Trieste, Italy) was erroneously omitted. The correct funding statement reads: **This work was supported by the Italian Ministry of Health, through the contribution given to the Institute for Maternal and Child Health IRCCS Burlo Garofolo, Trieste, Italy**.

The original version of this article has been updated.

