# Supplementary material for: Prevalence of white spot lesions in children and adolescents across Northern, Central and Southern Italy: a multicenter cross-sectional study
Source: Front Oral Health. 2026 Jun 30;7:1839880. doi: 10.3389/froh.2026.1839880 (PMC13365299; doi:10.3389/froh.2026.1839880)
Supplement: Supplementary file 1 [file Table1.docx]

Supplementary Material

**Table 1S**. Occurrence of white spot lesions related to sociodemographic and behavioral characteristics by Centers.

|  | **PALERMO**  **(N=404)** | **ROME**  **(N=467)** | **TRIESTE**  **(N=218)** | **OVERALL**  **(N=1089)** | **P-value** |
| --- | --- | --- | --- | --- | --- |
| **Occurrence of whitespots§** | |  |  |  |  |
| no | 137 (33.9%) | 170 (36.4%) | 118 (54.1%) | 425 (39.0%) | <0.001 |
| yes | 267 (66.1%) | 297 (63.6%) | 100 (45.9%) | 664 (61.0%) |  |
| **Age§** |  |  |  |  |  |
| less or equal to 8 years old | 173 (42.8%) | 92 (19.7%) | 94 (43.1%) | 359 (33.0%) | <0.001 |
| 9 years old | 57 (14.1%) | 121 (25.9%) | 19 (8.7%) | 197 (18.1%) |  |
| 10-11 years old | 89 (22.0%) | 163 (34.9%) | 47 (21.6%) | 299 (27.5%) |  |
| more or equal to 12 years old | 85 (21.0%) | 91 (19.5%) | 58 (26.6%) | 234 (21.5%) |  |
| **Sex§** |  |  |  |  |  |
| female | 193 (47.8%) | 225 (48.2%) | 107 (49.1%) | 525 (48.2%) | 0,992 |
| male | 211 (52.2%) | 242 (51.8%) | 111 (50.9%) | 564 (51.8%) |  |
| **BMI§§** |  |  |  |  |  |
| normal | 384 (95.0%) | 247 (52.9%) | 216 (99.1%) | 847 (77.8%) | 0,052 |
| overweight | 17 (4.2%) | 11 (2.4%) | 2 (0.9%) | 30 (2.8%) |  |
| obese | 3 (0.7%) | 3 (0.6%) | 0 (0%) | 6 (0.6%) |  |
| Missing | 0 (0%) | 206 (44.1%) | 0 (0%) | 206 (18.9%) |  |
| **Nationality§** |  |  |  |  |  |
| Italian | 374 (92.6%) | 354 (75.8%) | 192 (88.1%) | 920 (84.5%) | <0.001 |
| Not Italian | 30 (7.4%) | 113 (24.2%) | 26 (11.9%) | 169 (15.5%) |  |
| **Father occupational status§** | |  |  |  |  |
| working | 354 (87.6%) | 367 (78.6%) | 201 (92.2%) | 922 (84.7%) | <0.001 |
| not working | 29 (7.2%) | 90 (19.3%) | 6 (2.8%) | 125 (11.5%) |  |
| Missing | 21 (5.2%) | 10 (2.1%) | 11 (5.0%) | 42 (3.9%) |  |
| **Mother occupational status§** | |  |  |  |  |
| working | 275 (68.1%) | 288 (61.7%) | 150 (68.8%) | 713 (65.5%) | 0,0976 |
| not working | 124 (30.7%) | 177 (37.9%) | 65 (29.8%) | 366 (33.6%) |  |
| Missing | 5 (1.2%) | 2 (0.4%) | 3 (1.4%) | 10 (0.9%) |  |
| **Father marital status§** |  |  |  |  |  |
| in couple | 330 (81.7%) | 359 (76.9%) | 173 (79.4%) | 862 (79.2%) | 0,0449 |
| not in couple | 52 (12.9%) | 96 (20.6%) | 36 (16.5%) | 184 (16.9%) |  |
| Missing | 22 (5.4%) | 12 (2.6%) | 9 (4.1%) | 43 (3.9%) |  |
| **Mother marital status§** |  |  |  |  |  |
| in couple | 337 (83.4%) | 359 (76.9%) | 175 (80.3%) | 871 (80.0%) | 0,0654 |
| not in couple | 62 (15.3%) | 106 (22.7%) | 43 (19.7%) | 211 (19.4%) |  |
| Missing | 5 (1.2%) | 2 (0.4%) | 0 (0%) | 7 (0.6%) |  |
| **Father education§** |  |  |  |  |  |
| Compulsory education or lower | 125 (30.9%) | 145 (31.0%) | 63 (28.9%) | 333 (30.6%) | 0,956 |
| Higher education or degree | 259 (64.1%) | 313 (67.0%) | 145 (66.5%) | 717 (65.8%) |  |
| Missing | 20 (5.0%) | 9 (1.9%) | 10 (4.6%) | 39 (3.6%) |  |
| **Mother education§** |  |  |  |  |  |
| Compulsory education or lower | 142 (35.1%) | 239 (51.2%) | 47 (21.6%) | 428 (39.3%) | <0.001 |
| Higher education or degree | 255 (63.1%) | 226 (48.4%) | 170 (78.0%) | 651 (59.8%) |  |
| Missing | 7 (1.7%) | 2 (0.4%) | 1 (0.5%) | 10 (0.9%) |  |
| **Brush teeth§§** |  |  |  |  |  |
| never | 3 (0.7%) | 10 (2.1%) | 1 (0.5%) | 14 (1.3%) | <0.001 |
| after meals | 183 (45.3%) | 290 (62.1%) | 161 (73.9%) | 634 (58.2%) |  |
| occasionally | 218 (54.0%) | 167 (35.8%) | 56 (25.7%) | 441 (40.5%) |  |
| **Type of toothbrush§** |  |  |  |  |  |
| does not matter | 33 (8.2%) | 17 (3.6%) | 24 (11.0%) | 74 (6.8%) | <0.001 |
| manual | 312 (77.2%) | 346 (74.1%) | 141 (64.7%) | 799 (73.4%) |  |
| electric | 59 (14.6%) | 104 (22.3%) | 53 (24.3%) | 216 (19.8%) |  |
| **Braces§** |  |  |  |  |  |
| no | 385 (95.3%) | 381 (81.6%) | 202 (92.7%) | 968 (88.9%) | <0.001 |
| yes | 19 (4.7%) | 86 (18.4%) | 16 (7.3%) | 121 (11.1%) |  |
| **Sweetened pacifier§** |  |  |  |  |  |
| seldom/never | 383 (94.8%) | 367 (78.6%) | 210 (96.3%) | 960 (88.2%) | <0.001 |
| often/always | 7 (1.7%) | 55 (11.8%) | 7 (3.2%) | 69 (6.3%) |  |
| Missing | 14 (3.5%) | 45 (9.6%) | 1 (0.5%) | 60 (5.5%) |  |
| **Chewing-gum§** |  |  |  |  |  |
| seldom/never | 374 (92.6%) | 313 (67.0%) | 192 (88.1%) | 879 (80.7%) | <0.001 |
| often/always | 30 (7.4%) | 111 (23.8%) | 26 (11.9%) | 167 (15.3%) |  |
| Missing | 0 (0%) | 43 (9.2%) | 0 (0%) | 43 (3.9%) |  |
| **Fruit and vegetables intake§** | |  |  |  |  |
| twice a day or more | 226 (55.9%) | 308 (66.0%) | 87 (39.9%) | 621 (57.0%) | <0.001 |
| once a day | 178 (44.1%) | 159 (34.0%) | 131 (60.1%) | 468 (43.0%) |  |
| **Snacks intake§** |  |  |  |  |  |
| seldom/never | 156 (38.6%) | 326 (69.8%) | 63 (28.9%) | 545 (50.0%) | <0.001 |
| often/always | 248 (61.4%) | 105 (22.5%) | 155 (71.1%) | 508 (46.6%) |  |
| Missing | 0 (0%) | 36 (7.7%) | 0 (0%) | 36 (3.3%) |  |
| **Sweet beverages intake§** |  |  |  |  |  |
| seldom/never | 341 (84.4%) | 338 (72.4%) | 165 (75.7%) | 844 (77.5%) | 0,0423 |
| often/always | 62 (15.3%) | 90 (19.3%) | 53 (24.3%) | 205 (18.8%) |  |
| Missing | 1 (0.2%) | 39 (8.4%) | 0 (0%) | 40 (3.7%) |  |
| **Milk and diary products intake§** | |  |  |  |  |
| seldom/never | 90 (22.3%) | 344 (73.7%) | 39 (17.9%) | 473 (43.4%) | <0.001 |
| often/always | 312 (77.2%) | 88 (18.8%) | 179 (82.1%) | 579 (53.2%) |  |
| Missing | 2 (0.5%) | 35 (7.5%) | 0 (0%) | 37 (3.4%) |  |
| **Visits to a dentist in the year§** | |  |  |  |  |
| never | 146 (36.1%) | 174 (37.3%) | 86 (39.4%) | 406 (37.3%) | 0,882 |
| once or more | 258 (63.9%) | 293 (62.7%) | 132 (60.6%) | 683 (62.7%) |  |
| **Number of caries§§§** |  |  |  |  |  |
| Mean (SD) | 1.80 (2.85) | 1.06 (2.12) | 2.68 (3.82) | 1.66 (2.86) | <0.001 |
| Median [Min, Max] | 0 [0, 13.0] | 0 [0, 12.0] | 0.500 [0, 17.0] | 0 [0, 17.0] |  |

§p-value from the Pearson’s Chi-squared test ; §§p-value from the Fisher’s exact test; §§§p-value from the Wilcoxon rank sum test
